# Supplementary material for: Regulation of locomotor speed and selection of active sets of neurons by V1 neurons
Source: Nat Commun. 2019 May 22;10:2268. doi: 10.1038/s41467-019-09871-x (PMC6531463; doi:10.1038/s41467-019-09871-x)
Supplement: Supplementary file 1 — Supplementary Information [file 41467_2019_9871_MOESM1_ESM.pdf]

Supplementary Information

Regulation of locomotor speed and selection of active sets of neurons by V1 neurons

Kimura et al.

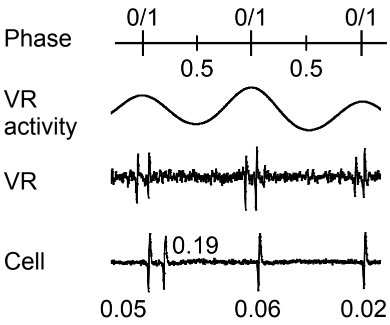

Supplementary Figure 1. Method for determining the firing timing of the recorded cell in each swimming cycle (related to Figure 1)

The VR recording was rectified and smoothened (VR activity). For the phase analysis of spike timings, the middle time point of a VR activity (above threshold) was assigned a phase value of 0, and that of the next VR activity was assigned a phase value of 1.

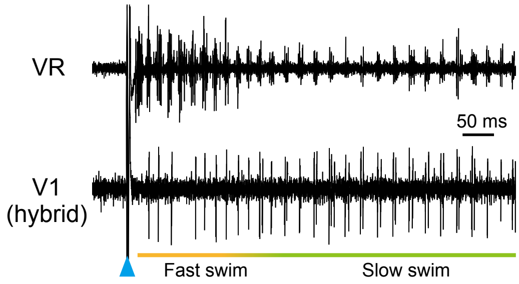

Supplementary Figure 2. Example of the recordings from the hybrid-type V1 neurons (related to Figure 1)

Example of simultaneous recordings between hybrid-type V1 neurons (loose-patch recording) and VR. Hybrid-type V1 neurons exhibited spiking activities during both fast and slow swimming.

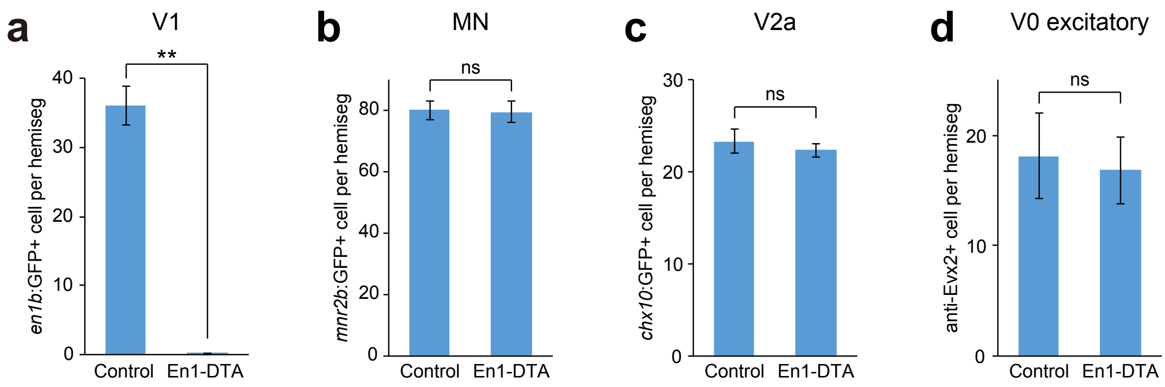

Supplementary Figure 3. Characterization of the spinal cord in V1-ablated fish (related to Figure 2)

a, Numbers of GFP-positive V1 neurons per hemi-segment in the triple transgenic fish (Tg [*en1b*:GFP], Tg[*en1b*:loxP-RFP-loxP-DTA], and Tg[*hoxa4a/9a*:Cre]) and control fish (without Cre) at 3 dpf. Control, 36.1 ± 2.8, En1-DTA, 0.1 ± 0.1. $P = 6.4 \times 10^{-9}$ (two-tailed t-test). **b**, Numbers of *mnr2b*:GFP-positive neurons (MNs) per hemi-segment in control and En1-DTA fish at 3 dpf. Control, 80.3± 3.1; En1-DTA, 79.6 ± 3.1. $P = 0.76$ (two-tailed t-test). **c**, Numbers of *chx10*:GFP-positive (V2a) neurons per hemi-segment in control and En1-DTA fish at 3 dpf. Control, 23.4 ± 1.4, En1-DTA, 22.4 ± 0.7. $P = 0.18$ (two-tailed t-test). **d**, Numbers of anti-Evx2 immunoreactive neurons (V0 excitatory neurons) per hemi-segment in control and En1-DTA fish at 2 dpf. Control, 18.1 ± 3.9; En1-DTA, 16.8 ± 3.1. $P = 0.57$ (two-tailed t-test). For **a-d**, four hemi-segments from five fish were examined. Data are mean ± s.d.. $^{**}P < 0.01$. ns, not significant.

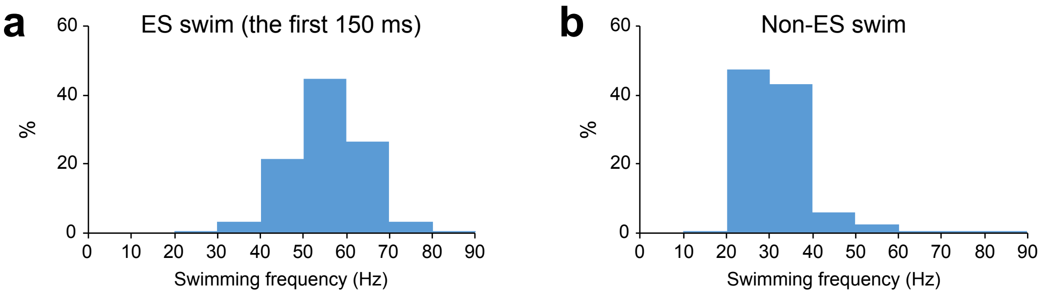

Supplementary Figure 4. Swimming frequencies during ES and Non-ES swim (related to Figure 2)

a, Histogram of swimming frequency during the initial phase of ES swim (within 150 ms after electrical stimulations). In total, 3821 swimming cycles from 73 fish were examined. **b**, Histogram of swimming frequency during Non-ES swim (spontaneously occurring swimming and illumination-induced swimming). In total, 42,002 swimming cycles from 73 fish were examined.

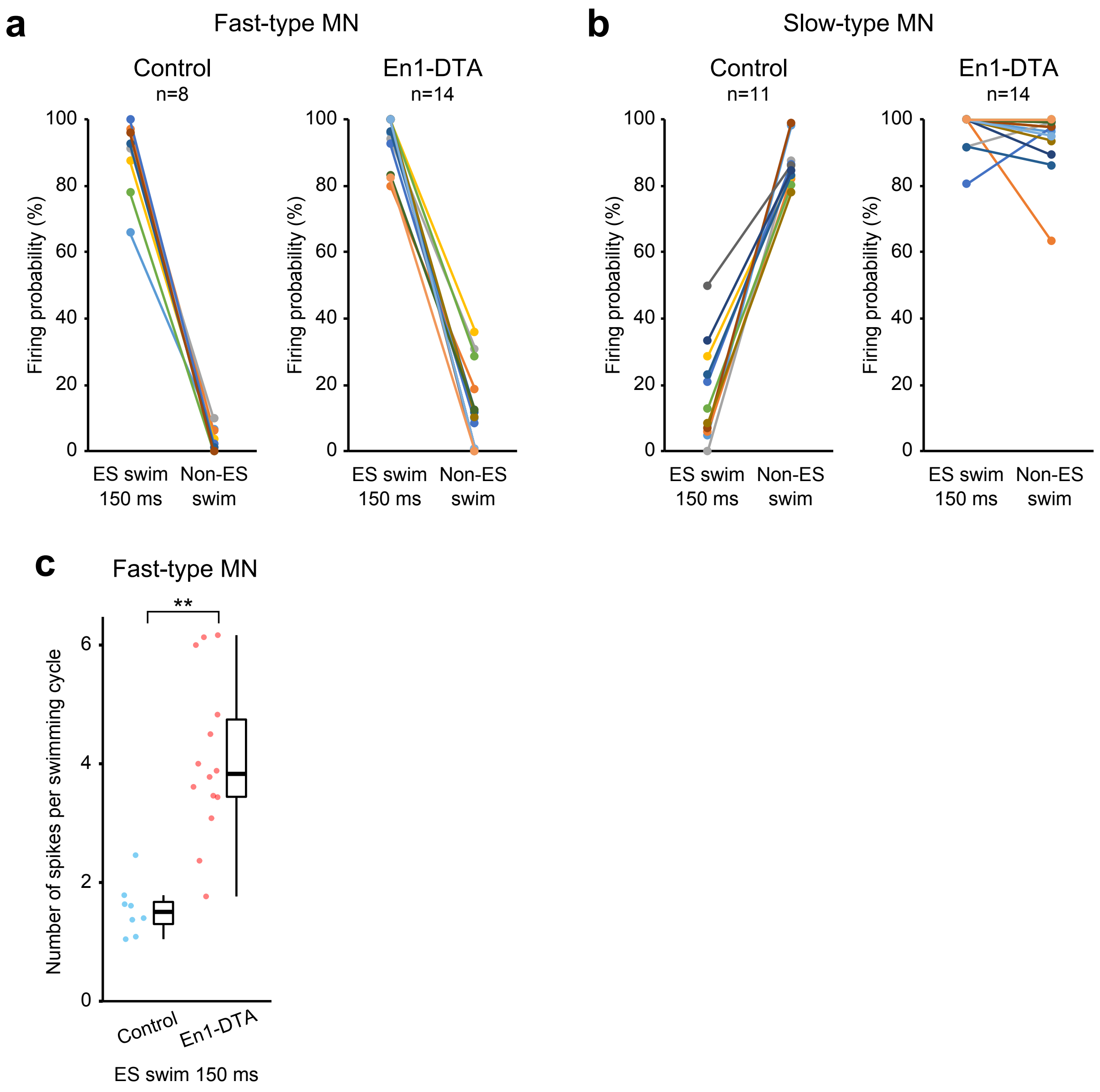

Supplementary Figure 5. Activity of fast-type and slow-type MNs in control and En1-DTA fish during fictive swimming (related to Figure 3)

a, Firing probability of fast-type MNs in each cycle during the initial phase of ES swim and Non-ES swim. **b**, Firing probability of slow-type MNs in each cycle during the initial phase of ES swim and Non-ES swim. **c**, Numbers of spikes per swimming cycle during the initial phase of ES swim in control (n=8) and En1-DTA fish (n=14). ***P* < 0.01 (Mann–Whitney U test, $P = 4.4 \times 10^{-5}$). Boxes represent the IQR between first and third quartiles and the line inside represents the median. Whiskers denote the lowest and highest values within 1.5 × IQR from the first and third quartiles, respectively.

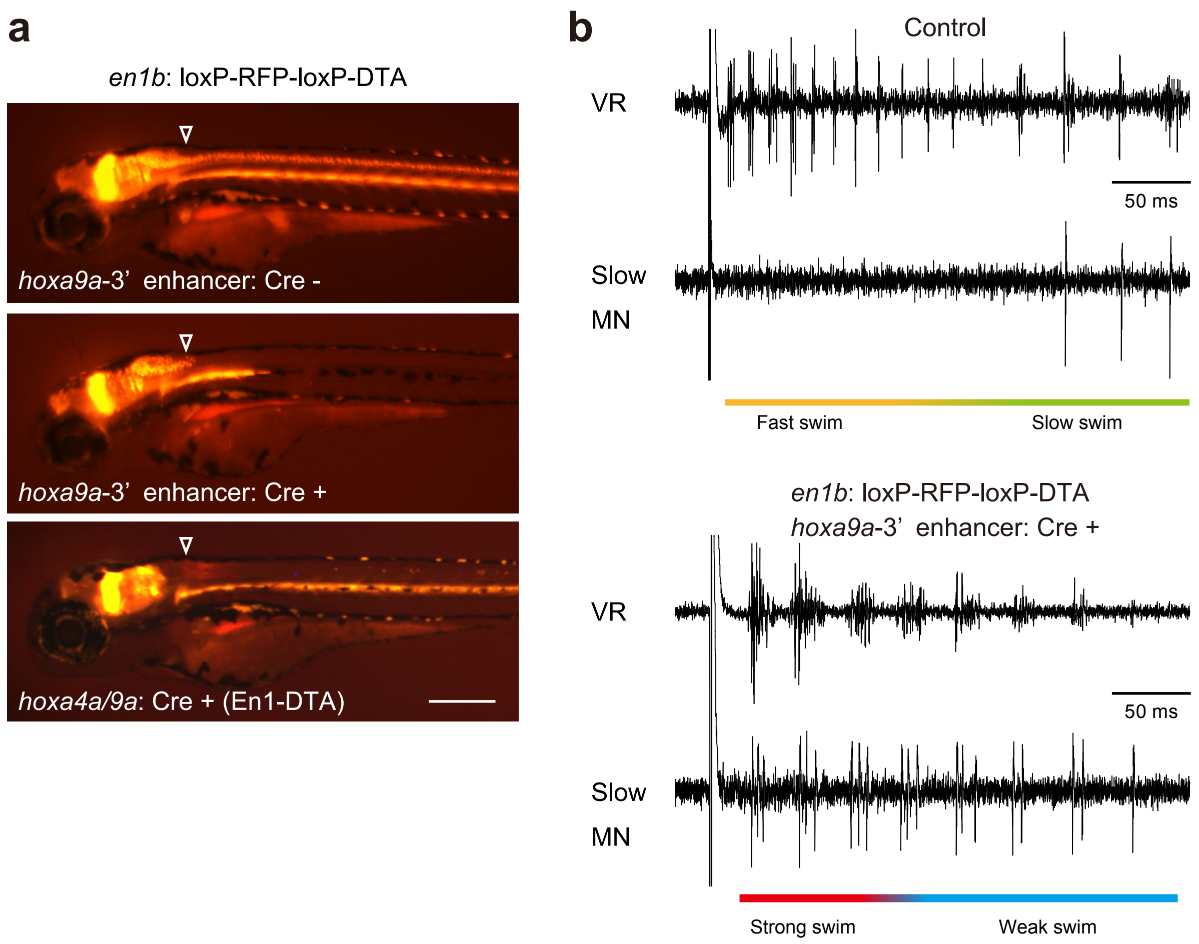

Supplementary Figure 6. Phenotype of the compound transgenic fish of Tg [*en1b*:loxP-RFP-loxP-DTA] and Tg[*hoxa9a*-3'enhancer:Cre] (related to Figures 2 and 3)

a, Fluorescent images of Tg[*en1b*:loxP-RFP-loxP-DTA] fish without a Cre driver (top panel), with Tg [*hoxa9a*-3'enhancer:Cre] (middle panel), and Tg[*hoxa4a/9a*:Cre] (bottom panel). Arrowheads indicate the boundary between the hindbrain and the spinal cord. Scale bar, 250 µm. **b**, VR and slow-type MN recordings during fictive swimming elicited by electrical stimulation in a control (top panel) and a compound transgenic fish of Tg[*en1b*:loxP-RFP-loxP-DTA] and Tg[*hoxa9a*-3'enhancer:Cre] (bottom panel).

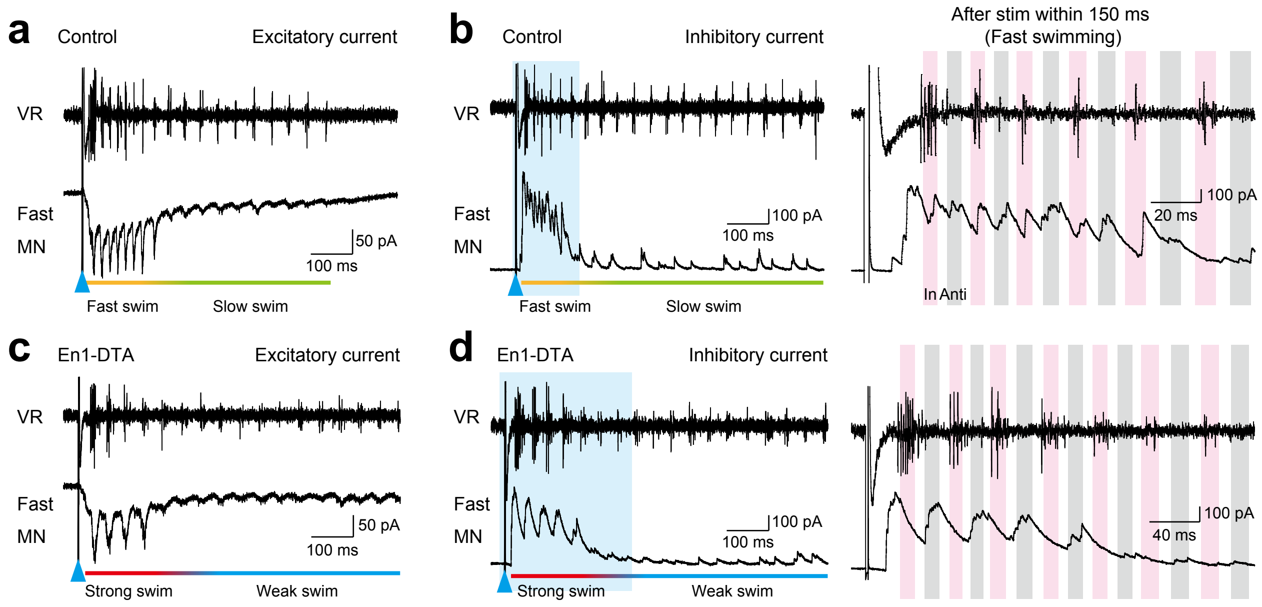

Supplementary Figure 7. Voltage-clamp recordings from fast-type MNs in control and En1-DTA fish during fictive swimming (related to Figure 4)

Voltage-clamp recordings from fast-type MNs in control and En1-DTA fish during fictive swimming. **a**, Example of simultaneous recordings between fast-type MNs and VR in control fish. The cell was held at -75 mV to reveal excitatory currents. **b**, Example of simultaneous recordings between fast-type MNs and VR in control fish. The cell was held at +10 mV to reveal inhibitory currents. The right panel shows the enlargement of the regions shaded in blue. **c**, Example of simultaneous recordings between fast-type MNs and VR in En1-DTA fish. The cell was held at -75 mV to reveal excitatory currents. **d**, Example of simultaneous recordings between fast-type MNs and VR in En1-DTA fish. The cell was held at +10 mV to reveal inhibitory currents. The right panel shows the enlargement of the regions shaded in blue. In total, five fast-type MNs were recorded in control fish, and six fast-type MNs were recorded in En1-DTA fish. We obtained similar results in these recordings.

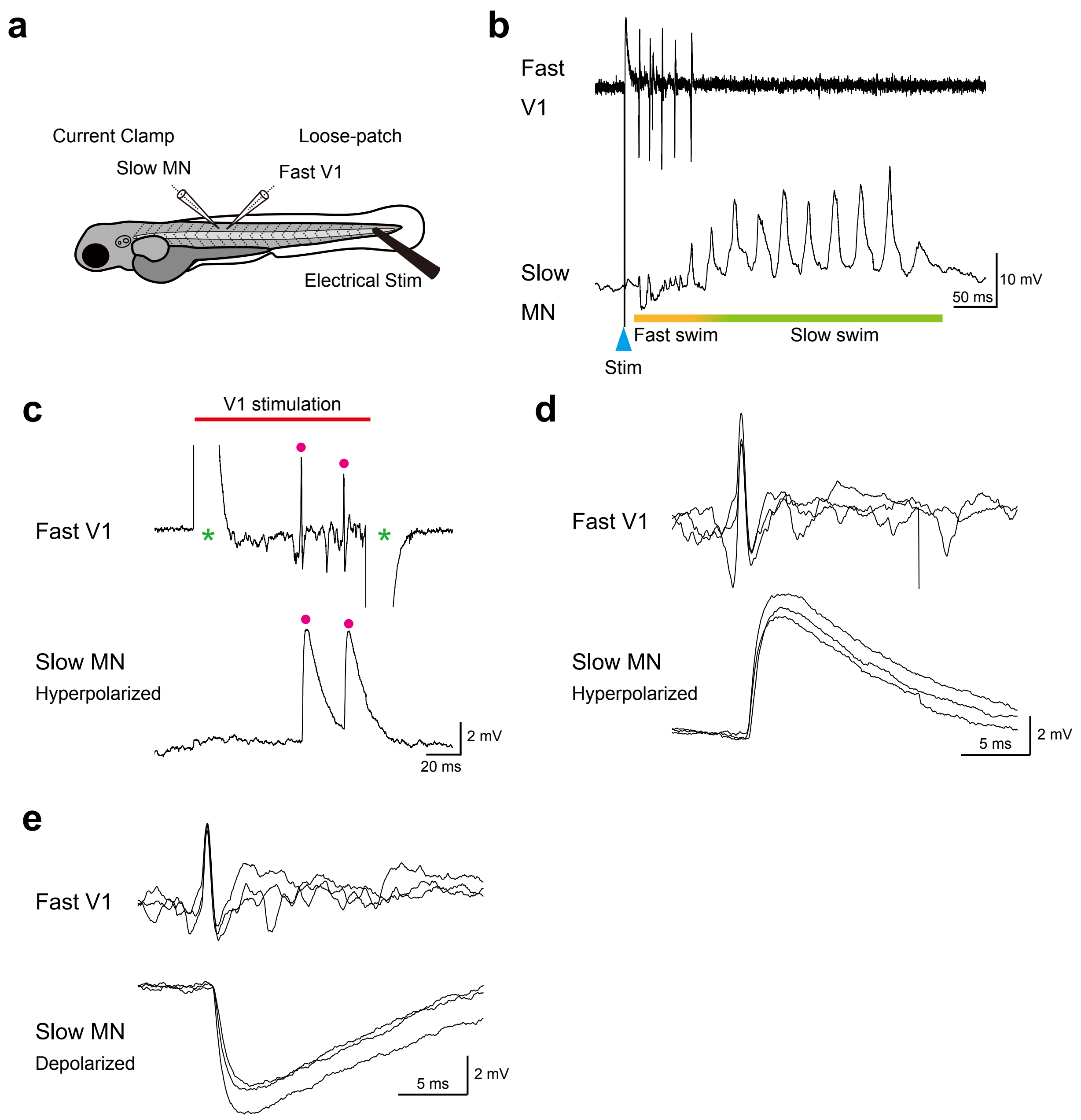

Supplementary Figure 8. Paired recordings between fast-type V1 neurons and slow-type MNs (related to Figure 4)

a, A schematic illustration of the simultaneous recordings between a V1 neuron (loose-patch) and a slow-type MN (whole cell, current clamp). **b**, An example of paired recordings between a fast-type V1 (top) and a slow-type MN (bottom) during fictive swimming elicited by electrical stimulation. **c**, Current application to a loose patch V1 neuron (red bar) fires the cell (red dots in the top panel). The gaps of the trace (green asterisks) are the artifacts caused by the current injection. The spikes are followed at short latency by depolarized IPSPs in the MN (red dots in the bottom panel). Note that the MN was hyperpolarized by a negative current injection. **d**, Three traces of V1 spikes and depolarized IPSP responses in the MN are superimposed. **e**, Same as **d**, but the experiment was performed under conditions in which the MN was depolarized by positive current injections. IPSP responses are apparent as hyperpolarizing.

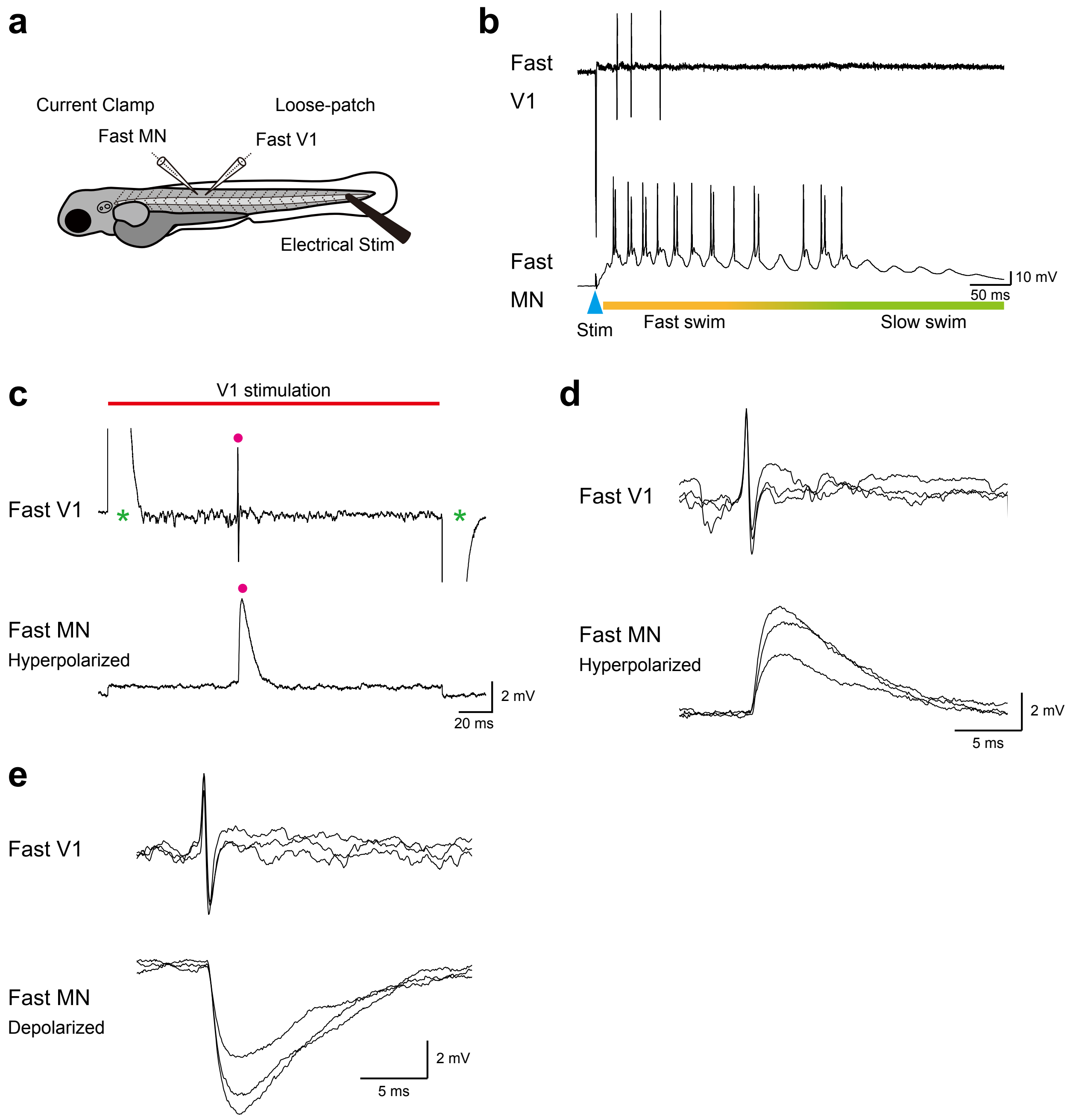

Supplementary Figure 9. Paired recordings between fast-type V1 neurons and fast-type MNs (related to Figure 4)

a, A schematic illustration of the experiment. **b**, An example of a paired recording between a fast-type V1 (top) and a fast-type MN (bottom) during fictive swimming elicited by electrical stimulation. **c**, Current application to a loose patch V1 neuron (red bar) fires the cell (red dot in the top panel). The gaps of the trace (green asterisks) are the artifacts caused by the current injection. The spikes are followed at short latency by depolarized IPSPs in the MN (red dot in the bottom panel). Note that the MN was hyperpolarized by a negative current injection. **d**, Three traces of V1 spikes and depolarized IPSP responses in the MN are superimposed. **e**, Same as **d**, but the experiment was performed under conditions in which the MN was depolarized by positive current injections. IPSP responses are apparent as hyperpolarizing.

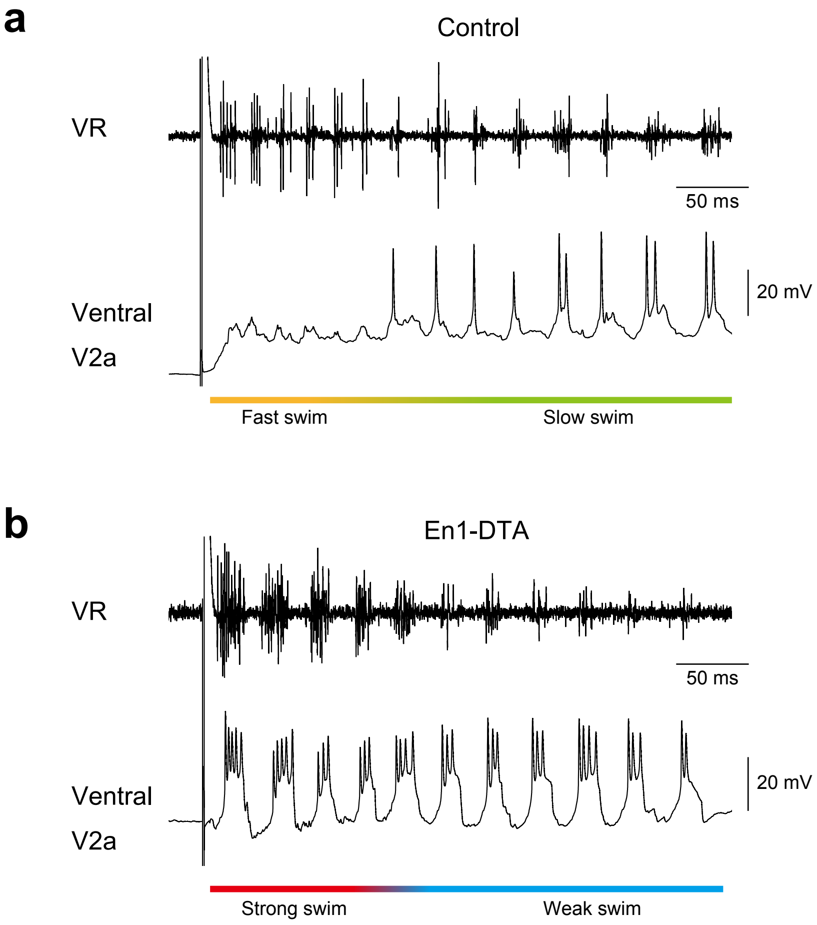

Supplementary Figure 10. Whole-cell recordings of ventrally located V2a neurons in control and En1-DTA fish during fictive swimming (related to Figure 5)

a, An example of simultaneous recordings between ventrally located V2a neurons (whole cell, current clamp) and VR in control fish. **b**, An example of simultaneous recordings between ventrally located V2a neurons (whole cell, current clamp) and VR in En1-DTA fish.

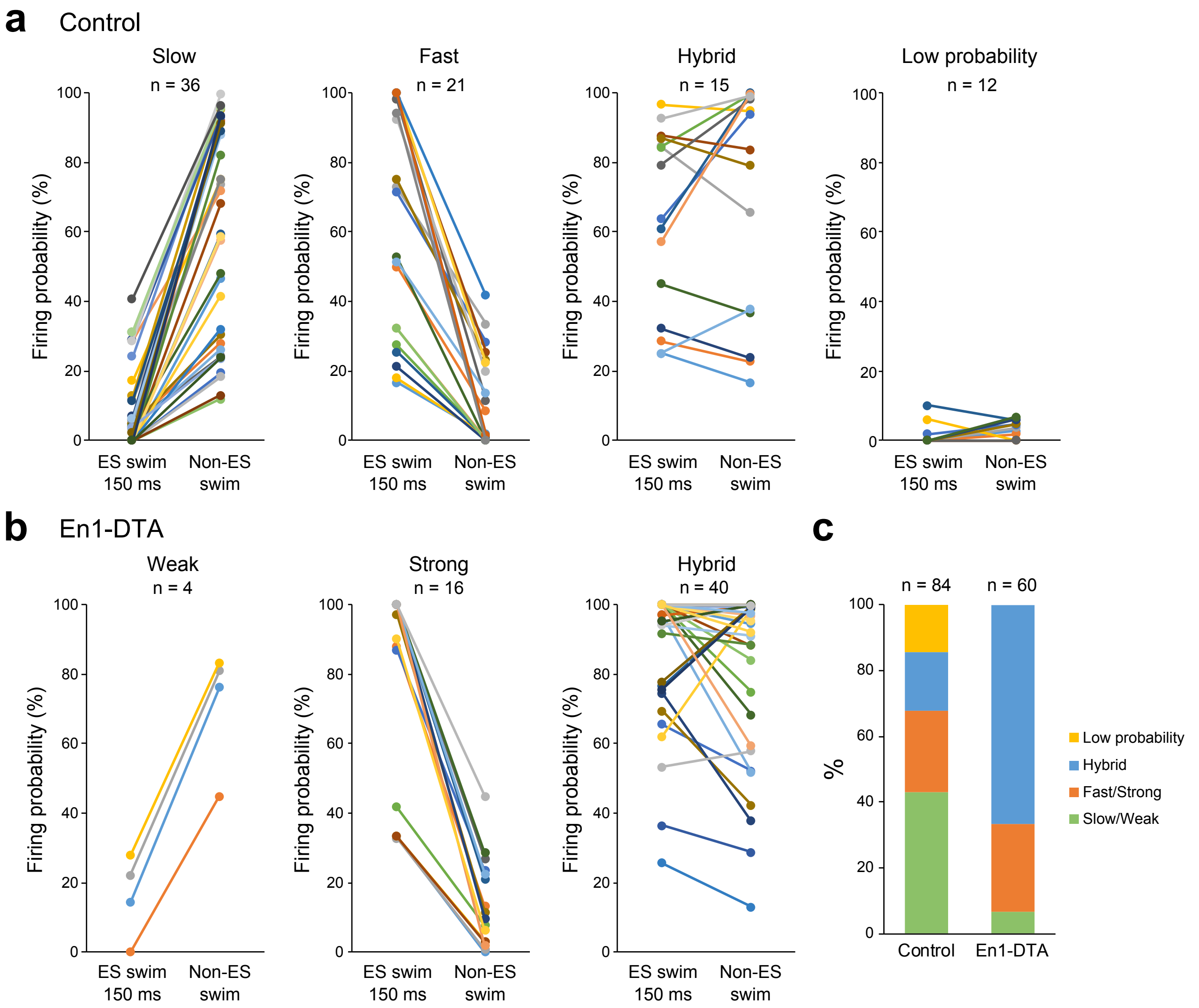

Supplementary Figure 11. Classification of ventrally located V2a neurons in control and En1-DTA fish based on their firing properties during fictive swimming (related to Figure 5)

Classification of ventrally located V2a neurons according to their firing properties during fast/strong swim (the initial phase of ES swim) and Non-ES swim, which mostly consists of slow/weak swim. **a**, Firing probability of ventrally located V2a neurons in each cycle during the initial phase of ES swim and Non-ES swim. In total, 84 neurons were recorded in control fish. Thirty-six neurons were classified as "slow" type. In these neurons, the value of the firing probability (percentage) during Non-ES swim was more than double that during ES swim (the first 150 ms) (leftmost panel). Twenty-one neurons were classified as "fast" type as per the criteria described above (middle left). Fifteen neurons were classified as "hybrid" type. In these neurons, the difference was within the doubled value (middle right). Twelve neurons were classified as "low-probability" type. In these neurons, firing probability was below 10% during both the ES and Non-ES swim (rightmost panel). **b**, Firing probability of ventrally located V2a neurons in each cycle during the initial phase of ES swim and Non-ES swim. In total, 60 neurons were recorded in En1-DTA fish. Four neurons were classified as "weak" type (left). Sixteen neurons were classified as "strong" type (middle). Forty neurons were classified as "hybrid" type (right). **c**, Summary of the classification of ventrally located V2a neurons in control and En1-DTA fish. Note that the slow/weak-type of neurons (green) was greatly reduced in En1-DTA fish.

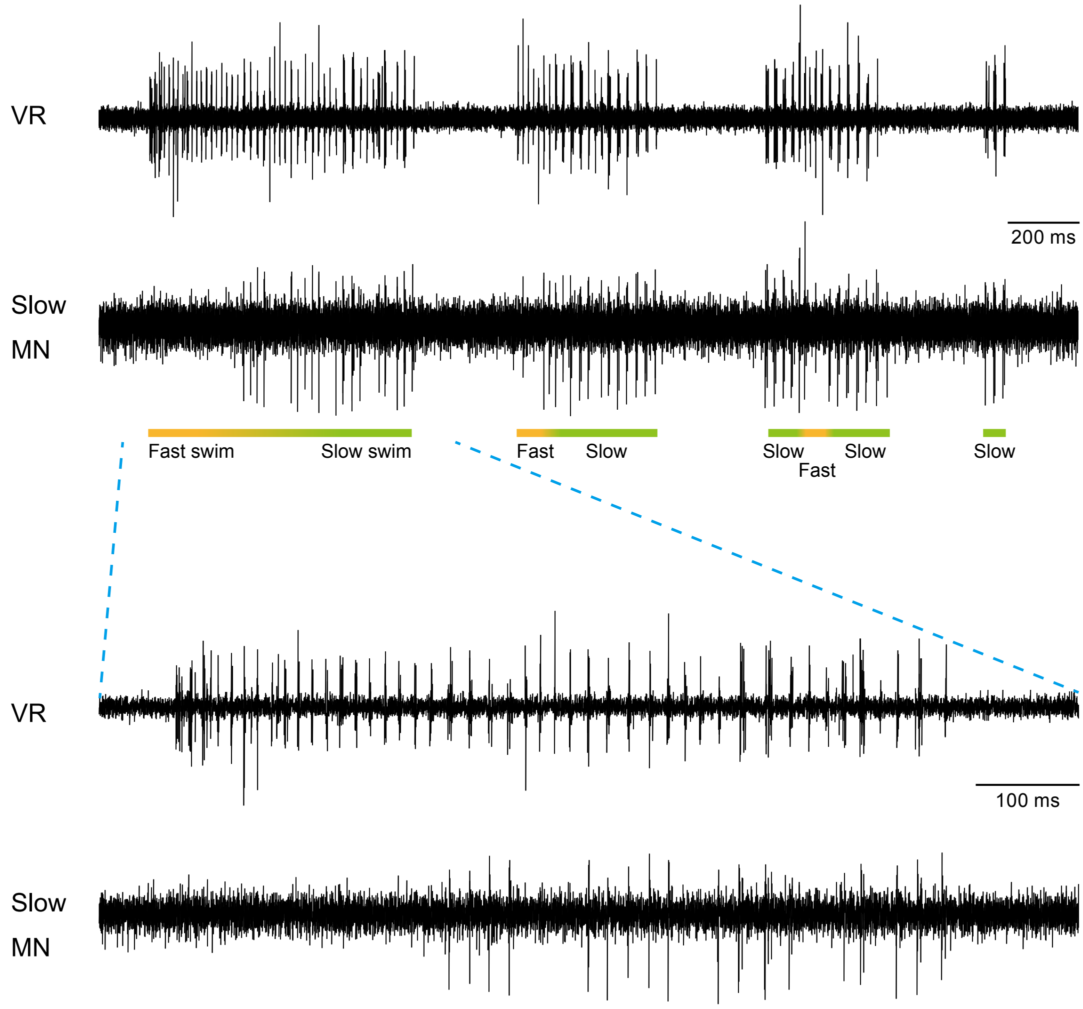

Supplementary Figure 12. Activities of slow-type MNs during fictive non-ES swim (related to Figure 6)

For each panel (top and bottom), the top trace shows the VR recording, while the bottom trace shows the extracellular recording of a slow-type MN. The bottom panel is a magnified view. Spikes in the slow-type MN tend to be absent during fast (short-cycle period) swim.
