# Supplementary material for: Regulation of locomotor speed and selection of active sets of neurons by V1 neurons
Source: Nat Commun. 2019 May 22;10:2268. doi: 10.1038/s41467-019-09871-x (PMC6531463; doi:10.1038/s41467-019-09871-x)
Supplement: Supplementary file 4 — Description of Additional Supplementary Files [file 41467_2019_9871_MOESM4_ESM.pdf]

Description of Additional Supplementary Files

File Name: Supplementary Movie 1

Description: **Evoked swimming upon sudden tactile stimuli in control and En1-DTA larvae**

Upon sudden-touch stimulation, the swimming caused by large-amplitude muscular contractions was observed in both control (top) and En1-DTA fish (bottom) at 3 dpf. In En1-DTA fish, the duration of each bending, including the escape bend, was extremely prolonged.
